# Unusual Association of Inverse Retinitis Pigmentosa, Scleromalacia, and Neovascular Glaucoma

**DOI:** 10.4274/tjo.galenos.2020.07573

**Published:** 2020-04-29

**Authors:** Ayşe Gül Koçak Altıntaş, Çağrı İlhan, Mehmet Çıtırık

**Affiliations:** 1 University of Helath Science, Ulucanlar Göz Training and Research Hospital, Clinic of Ophthalmology, Ankara, Turkey; 2 Hatay State Hospital, Clinic of Ophthalmology, Hatay, Turkey

**Keywords:** Anti-vascular endothelial growth factor, inverse retinitis pigmentosa, intracameral bevacizumab, intraocular inflammation, neovascular glaucoma

## Abstract

A 31-year-old woman with inverse retinitis pigmentosa presented with severe ocular pain and ingrained visual loss. Biomicroscopy revealed a large scleromalacia area above the superior limbus, minimal Descemet’s membrane folds, aqueous flare, rubeosis iridis, and mature cataract. Intraocular pressure was 39 mmHg, and the clinical picture was consistent with neovascular glaucoma. After immediate medication to reduce ocular discomfort, an anterior chamber bevacizumab injection was performed. At 1 week post-injection, the rubeosis iridis had largely regressed and intraocular pressure was 21 mmHg. At post-injection 1 month, antiglaucomatous medication was discontinued because intraocular pressure was stable. Clear cornea, normal anterior chamber depth, and mature cataract were seen via biomicroscopy, and increased axial length with no significant change in posterior segment echogenicity were observed on ultrasonography. Three years after the single dose of bevacizumab, neovascularization was not seen in either the anterior chamber angle or on the iris surface, and intraocular pressure remained within normal range. The most important aspect of this case report is that it is the first to show an unusual association between neovascular glaucoma, scleromalacia, and inverse retinitis pigmentosa.

## Introduction

Retinitis pigmentosa (RP) is a heterogeneous group of inherited disorders characterized by photoreceptor and retinal pigment epithelium (RPE) abnormalities. It can be inherited in an autosomal dominant, autosomal recessive, or X-linked manner, and over 40 genes are associated with this group of retinal dystrophies.^1^ Typical clinical symptoms are night blindness, reduced central vision, and visual field constriction. Mid-peripheral pigment migration, vascular attenuation, and disc pallor are the classical triad of retinal findings of RP.^2^ Primary open-angle glaucoma, early-onset senile cataract, and cystoid macular edema are relatively common complications of the disease, which accelerates permanent visual loss.^2^

Inverse RP is a rare form of RP that initially affects photoreceptors in the macula, causing significant visual impairment at very early stages of presentation. Autosomal recessive inheritance has been suggested. Many authors agree that this rare form of RP may correspond to cone-rod dystrophy with macular hyperpigmentation. However, diagnosis is difficult, and other inherited retinal disorders, such as Leber’s congenital neurosis, progressive cone-rod dystrophy, and central areolar choroidal sclerosis should be excluded.^3^

In this case report, we present a patient with an unusual association of inverse RP, scleromalacia, and neovascular glaucoma (NVG), which was treated with an intracameral anti-vascular endothelial growth factor (VEGF). To the best of our knowledge, this is the first report to show an association between RP and anterior segment neovascularization and scleromalacia.

## Case Report

**Figure 1:**
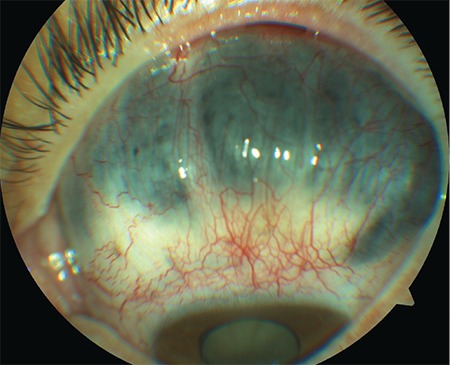
Large scleromalacia area over the superior limbus

A 31-year-old woman presented with ocular pain and ingrained visual loss in her left eye. The best corrected visual acuity (BCVA) was counting fingers at 1 m in the right eye and light perception (LP) with projection in the left eye. Biomicroscopy revealed a 2+ cataract in the right eye and a large scleromalacia area over the superior limbus, minimal Descemet’s membrane folds, aqueous flare, rubeosis iridis, and 4+ cataract in the left eye (Figure 1). Intraocular pressures (IOP) were 20 mmHg and 39 mmHg in the right and left eye, respectively. Waxy pallor optic disc, attenuation in retinal arterioles, and hyper- and hypo-pigmented RPE changes forming ‘bone spicules’ scattered in the posterior pole up to the equator, along with pigment clumping in both macular zones were seen, which are the classic clinical findings of inverse RP.

**Figure 2:**
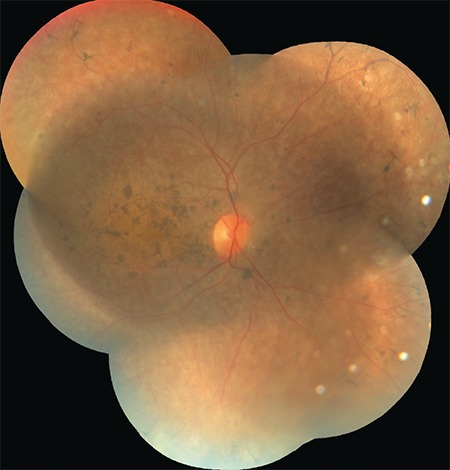
Color fundus photograph of the right retina

**Figure 3:**
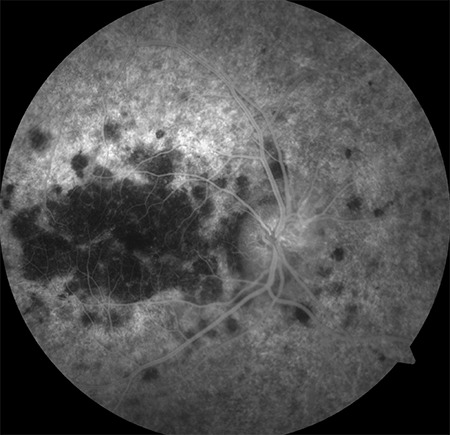
Red-free fundus photograph of the right retina

**Figure 4:**
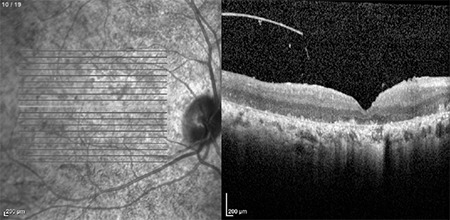
Optical coherence tomography of the right retina

**Figure 5:**
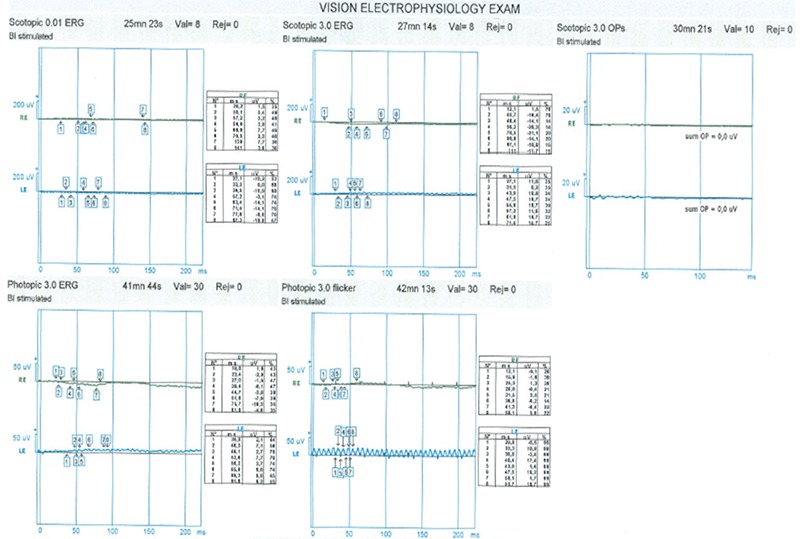
Electroretinography of the right retina

Clear color and red-free fundus photographs of the right retina could be taken after pupil dilation with 1% tropicamide due to relatively dense cataract (Figures 2 and 3). Fundus fluorescein angiography (FFA) showed central hypofluorescence due to contrasting blockage in areas with pigment accumulation and patchy hyperfluorescence due to window defects in the RPE atrophy areas. On optical coherence tomography (OCT), loss of photoreceptors, external limiting membrane, ellipsoid zone, and discontinuity of the outer retinal structures were seen (Figure 4). In B-mode ultrasonography of the left eye, the retina was attached, and there was no increase of echogenicity in the vitreous cavity. The axial length of the globe was detected as 24.58 mm on A-mode ultrasonography, which was nearly 2 mm longer than in the right eye. Electroretinography revealed significantly decreased amplitudes in all five recordings (rod, maximum, oscillatory, cone, and flicker) (p<0.05). The amplitude of b-waves in the rod, maximum, and cone responses was also reduced. Oscillatory P2 peak and flicker amplitudes also showed reduction in the recordings (Figure 5).

The patient had been receiving a combination of topical brinzolamide+0.5% timolol (1%), 0.2% brimonidine, and oral 250 mg acetazolamide 4 times a day for a week. To reduce ocular discomfort, 600 cc intravenous mannitol was administered. IOP was then only slightly reduced to 30 mmHg, and her ocular discomfort did not improve significantly. To induce regression of the rubeosis iridis, 1.25 mg/0.05 mL bevacizumab was injected into the anterior chamber. At 1 week post-injection, the rubeosis iridis was seen to have largely regressed and IOP was 21 mmHg.

The patient was instructed to continue using topical antiglaucomatous medication. At 1 month post-injection, the rubeosis iridis was observed to have completely regressed, IOP was 16 mmHg, and the patient had no ocular discomfort except low vision. All topical drugs were terminated and cataract extraction was suggested; however, the patient refused.

The patient was born from a consanguineous marriage of first-degree cousins and her siblings had also had RP, while the parents had no ocular disease. There was no history of past ocular diseases, surgery, trauma, systemic diseases, or drug use. There were no previous ocular inflammation or infection-related findings including corneal leukoma, corneal vascularization, lipid keratopathy, seclusion pupil, or sectoral iris atrophy in anterior segment examination. There was no any sign of intraocular or orbital tumor-suspected lesion in ocular B-scan ultrasonography or orbital computed tomography. To investigate the presence of scleromalacia or rubeosis iridis-related inflammatory or ischemic conditions, the patient was referred to an experienced internal disease specialist. After a physical examination, laboratory, and radiological investigations, no systemic disease was found, including diabetes mellitus, systemic lupus erythematosus, immune deficiency, leukemia/lymphoma, or plasma cell diseases. The absence of carotid artery stenosis was reported by the radiologist. No additional risk factor for presence of scleromalacia or NVG was found in any of these extensive evaluations.

Three years later, her IOP levels were 13 mmHg and 16 mmHg without medication. The anterior segment of the right eye was completely normal except for the 2+ cataract, while the left eye exhibited clear cornea, normal anterior chamber depth, an irregular moth-eaten atrophic iris, and minimal pupillary light response, along with 4+ cataract. No neovascularization in the anterior chamber angle was seen in either eye by gonioscopy. Posterior segment findings of the right eye were stable on fundoscopy, FFA, and OCT, while the left eye retina was attached without increased echogenicity on B-mode ultrasonography. There were still no findings of any diagnosed systemic inflammatory or ischemic diseases and no use of any drugs. Written informed consent was obtained from the patient to use her medical records for academic purposes.

## Discussion

The presence of NVG and rubeosis iridis in a case of inverse RP was very unexpected. The most common cause of NVG is retinal vein occlusion, especially an ischemic type central retinal vein occlusion.^4^ To the best of our knowledge, there is only one report in the literature that mentions ischemic central retinal vein occlusion in a patient with RP.^5^ In this case, possible past ischemic central retinal vein occlusion may have been the cause of rubeosis iridis and NVG. Even in this hypothesis, NVG can be explained with ischemic central retinal vein occlusion; however, the reason for dense cataract presentation in the same eye in a 31-year-old patient could not be explained. While early-onset senile cataracts occur in RP, this patient was very young and the cataracts in both eyes were significantly asymmetrical.^2^ The exact reason for progression of pathological new vessels could not be determined because the posterior segment of the globe was covered by cataract. This is the most significant disadvantage of this study, that the definitive cause of rubeosis iridis and NVG in a patient with RP could not be clarified.

Some reports have mentioned the coexistence of retinal neovascularization and RP.^6,7,8^ One of these mentioned the presence of NVG in RP.^8^ However, the patient in that report had multiple ocular comorbidities including cataract, vitreous hemorrhage, subretinal exudation, and diffuse posterior pole edema.^8^ The patient had multiple ocular surgeries before NVG, including bilateral lens extraction and retinal detachment surgery on the fellow eye.^8^ The patient underwent multiple systemic steroid treatments, and there was no comprehensive investigation about NVG-related systemic inflammatory or ischemic conditions.^8^ Therefore, the relationship between NVG and RP was unclear and disputable in that case because there were many NVG-related risk factors other than RP. In contrast, NVG in our RP patient, who had no history of ocular surgery, was not associated with any other known ocular condition. In addition, the patient had no systemic NVG-related condition or history of steroid use. Therefore, it can still be argued that this case is the first presentation of NVG in a patient with RP.

Recurrent anterior uveitis attacks can be observed in RP; however, they generally do not cause neovascularization. Extensive ocular inflammatory diseases, including choroiditis, panuveitis, or scleritis can be reasons for general ocular ischemia and scleromalacia.^9^ These diseases may explain unilateral NVG and cataract, and some may result in scleromalacia. A weak point of this hypothesis is there was no history of severe ocular inflammatory diseases or clinical findings of past uveitis, such as synechiae, keratic precipitates, pigment on the anterior lens capsule, etc. The exact mechanism of anterior segment neovascularization in this case remains unclear and it should not be completely ruled out that the relationship between RP, scleromalacia, and NVG may be just a coincidence.

Bevacizumab is a humanized monoclonal antibody that affects all VEGF isoforms. Its injection into the anterior chamber can provide a rapid regression of neovascularization on both the iris surface and anterior chamber angle.^10^ In the current case, IOP decreased to a normal level 1 week after the intracameral anti-VEGF injection. Throughout the 3-year follow-up, stable IOP was provided with a single injection, without additional medication. A 3-year follow-up period is sufficient for the treatment of NVG, and as far as we know, this is the longest period showing the efficacy of a single dose of intracameral bevacizumab. In addition, to the best of our knowledge, this is the first case of a patient with scleromalacia receiving intracameral bevacizumab injection without any long-term complications. Although the pathogenesis of the anterior chamber neovascularization could not be fully explained, one of most important aspects of this study is that this is the first report to show the association of NVG, scleromalacia, and RP.

## References

[1] Ferrari S, Di Iorio E, Barbaro V, Ponzin D, Sorrentino FS, Parmeggiani F, et al (2011). Retinitis pigmentosa: Genes and disease mechanisms. Curr Genomics..

[2] Davies EC, Pineda R 2nd (2017). Cataract surgery outcomes and complications in retinal dystrophy patients. Can J Ophthalmol..

[3] Díez-Cattini GF, Ancona-Lezama DA, Valdés-Lara C, Morales-Cantón V (2017). The unusual association of inverse retinitis pigmentosa and Fuchs’ heterochronic iridocyclitis. Int J Retina Vitreous..

[4] Hayreh SS, Rojas P, Podhajsky P, Montague P, Woolson RF (1983). Ocular neovascularization with retinal vascular occlusion-III. Incidence of ocular neovascularization with retinal vein occlusion. Ophthalmology..

[5] Paxhia MJ, Ting TD, Fekrat S (2001). Ischemic central retinal vein occlusion and retinitis pigmentosa: lower risk of neovascularization?. Retina.

[6] Butner RW (1984). Retinitis pigmentosa and retinal neovascularization: a case report. Ann Ophthalmol.

[7] Preethi S, Rajalakshmi AR (2015). Proliferative diabetic retinopathy in typical retinitis pigmentosa. BMJ Case Rep.

[8] Uliss AE, Gregor ZJ, Bird AC (1986). Retinitis pigmentosa and retinal neovascularization. Ophthalmology..

[9] Pichi F, Sarraf D, Arepalli S, Lowder CY, Cunningham ET Jr, Neri P, Albini TA, Gupta V, Baynes K, Srivastava SK (2017). The application of optical coherence tomography angiography in uveitis and inflammatory eye diseases. ProgRetin Eye Res..

[10] Altintas AG, Arifoglu HB, Tutar E, Koklu G, Ozcan PY (2012). Effect on anterior chamber bevacizumab injection combined with seton implantation in treatment of rubeosis iridis in neovascular glaucoma Cuban Ocul. Toxicol.

